# Validation of [^18^F]FLT as a perfusion-independent imaging biomarker of tumour response in EGFR-mutated NSCLC patients undergoing treatment with an EGFR tyrosine kinase inhibitor

**DOI:** 10.1186/s13550-018-0376-6

**Published:** 2018-03-27

**Authors:** R. Iqbal, G. M. Kramer, V. Frings, E. F. Smit, O. S. Hoekstra, R. Boellaard, on behalf of the QuIC-ConCePT Consortium

**Affiliations:** 1 Department of Radiology & Nuclear Medicine, VU University Medical Center, PO Box 7057, 1007 MB Amsterdam, The Netherlands; 2 Department of Pulmonology, VU University Medical Center, Amsterdam, The Netherlands

**Keywords:** Positron emission tomography (PET), Non-small cell lung cancer (NSCLC), [^18^F]FLT, Perfusion, Tyrosine kinase inhibitors (TKIs)

## Abstract

**Background:**

3′-Deoxy-3′-[^18^F]fluorothymidine ([^18^F]FLT) was proposed as an imaging biomarker for the assessment of in vivo cellular proliferation with positron emission tomography (PET). The current study aimed to validate [^18^F]FLT as a perfusion-independent PET tracer, by gaining insight in the intra-tumoural relationship between [^18^F]FLT uptake and perfusion in non-small cell lung cancer (NSCLC) patients undergoing treatment with a tyrosine kinase inhibitor (TKI).

Six patients with metastatic NSCLC, having an activating epidermal growth factor receptor (EGFR) mutation, were included in this study. Patients underwent [^15^O]H_2_O and [^18^F]FLT PET/CT scans at three time points: before treatment and 7 and 28 days after treatment with a TKI (erlotinib or gefitinib). Parametric analyses were performed to generate quantitative 3D images of both perfusion measured with [^15^O]H_2_O and proliferation measured with [^18^F]FLT volume of distribution (V_*T*_). A multiparametric classification was performed by classifying voxels as low and high perfusion and/or low and high [^18^F]FLT V_*T*_ using a single global threshold for all scans and subjects. By combining these initial classifications, voxels were allocated to four categories (low perfusion-low V_*T*_, low perfusion-high V_*T*_, high perfusion-low V_*T*_ and high perfusion-high V_*T*_).

**Results:**

A total of 17 perfusion and 18 [^18^F]FLT PET/CT scans were evaluated. The average tumour values across all lesions were 0.53 ± 0.26 mL cm^− 3^ min^− 1^ and 4.25 ± 1.71 mL cm^− 3^ for perfusion and [^18^F]FLT V_*T*_, respectively. Multiparametric analysis suggested a shift in voxel distribution, particularly regarding the V_*T*_: from an average of ≥ 77% voxels classified in the “high V_*T*_ category” to ≥ 85% voxels classified in the “low V_*T*_ category”. The shift was most prominent 7 days after treatment and remained relatively similar afterwards. Changes in perfusion and its spatial distribution were minimal.

**Conclusion:**

The present study suggests that [^18^F]FLT might be a perfusion-independent PET tracer for measuring tumour response as parametric changes in [^18^F]FLT uptake occurred independent from changes in perfusion.

**Trial registration:**

Nederlands Trial Register (NTR), NTR3557. Registered 2 August 2012

**Electronic supplementary material:**

The online version of this article (10.1186/s13550-018-0376-6) contains supplementary material, which is available to authorized users.

## Background

Non-small cell lung cancer (NSCLC) is a heterogeneous disease, comprising more than 85% of all the lung cancer cases [1]. Patients with NSCLC are often diagnosed at an advanced stage when curative treatment is no longer possible [2]. However, diagnostic advances have shown the importance of specific biological features in NSCLC, which might be used as potential therapeutic targets for treatment of these tumours [1]. One of these features includes genetic alterations of the epidermal growth factor receptor (EGFR) [3, 4].

EGFR is a transmembrane receptor with cytoplasmic kinase activity. The receptor is involved in several cellular processes such as proliferation, angiogenesis, metastasis and apoptosis [5, 6]. Patients with an activating EGFR mutation undergo continuous stimulation of the EGFR signalling pathway which results in increased proliferation, angiogenesis, metastasis and decreased apoptosis [7]. Specific targeting drugs, including tyrosine kinase inhibitors (TKIs) such as erlotinib and gefitinib, have been developed to prevent the downstream effects of the EGFR pathway [3, 8, 9].

Proliferation of (tumour) cells, one of the effects associated with the EGFR signalling pathway, can be visualised and evaluated with positron emission tomography (PET) using 3′-deoxy-3′-[^18^F]fluorothymidine ([^18^F]FLT) [3, 10–13]. Several studies have been performed to assess the correlation between [^18^F]FLT uptake and tumour cell proliferation as assessed with Ki-67 immunostaining. However, the results have been conflicting: while some studies confirm a positive correlation between [^18^F]FLT uptake and Ki-67 immunostaining [14, 15], others show a negative correlation between both. Various biological explanations, including differences in [^18^F]FLT metabolism, expression and activity of thymidine kinase and adenosine triphosphate levels (this is a cofactor for tyrosine kinase activity), have been proposed to explain these conflicting findings [16, 17]. Besides these, other biological features of the tumour such as its vascularity and its perfusion, might also cause variation in [^18^F]FLT uptake.

Perfusion is required for the delivery of nutrients, e.g. oxygen and glucose, necessary for further growth and progression of the tumour [13, 18]. Moreover, perfusion is also required for the delivery and uptake of the PET tracer in the tumour [19]. In an ideal situation, tracer delivery and tumour uptake are not perfusion dependent. However, it might be possible that perfusion affects tracer uptake: low perfused tumour lesions might show low tracer uptake due to limited supply of the tracer to the lesion. This might lead to an underestimation of the specific cellular process as assessed by the tracer, in this case, an underestimation of tumour proliferation as identified with [^18^F]FLT PET.

We hypothesise that [^18^F]FLT uptake will be decreased in low perfused tumour lesions. Furthermore, as during this study patients will be treated with a TKI, which has an inhibiting effect on tumour angiogenesis and tumour perfusion, it can theoretically be hypothesised that during/after treatment lesions will be low vascularized and perfused. This might limit supply of the [^18^F]FLT tracer to the lesion, leading to decreased [^18^F]FLT uptake. The aim of the current study was to investigate whether [^18^F]FLT is a perfusion-independent PET tracer by gaining insight in the intra-tumoural relationship between [^18^F]FLT uptake and perfusion and their changes in EGFR mutated NSCLC patients undergoing treatment with erlotinib or gefitinib.

## Methods

### Patients

Patients (*n* = 10) diagnosed with stage IV NSCLC and an activating EGFR mutation were included in this prospective clinical study. Further inclusion criteria were a primary tumour diameter of ≥ 3 cm and work-up for treatment with erlotinib or gefitinib. Exclusion criteria comprised pregnancy or lactation, inability to remain supine for 90 min, body weight > 100 kg and metal implants (for example pacemakers). Patients were recruited at six different medical centres in The Netherlands. All scans were performed at the VU University Medical Center. The study was included in the Dutch trial register (trialregister.nl, identification number NTR3557). Prior to inclusion, each patient provided written informed consent in accordance with the regulations of the Medical Ethics Review Committee of the VU University Medical Center.

### Study rationale

Several studies have evaluated [^18^F]FLT as a proliferation biomarker [3, 10, 11, 14]. However, most of these studies have not taken potential perfusion effects into account [10, 11, 14]. During this study, patients will undergo treatment with a TKI, which also intervenes with the vascular endothelial growth factor (VEGF) pathway. This treatment has an inhibiting effect on tumour angiogenesis and its perfusion [20, 21]. Therefore, during this study, it might be possible to assess perfusion changes and to evaluate its effect on [^18^F]FLT uptake. As compared to our previous study (3) where we have investigated the relationship between [^18^F]FLT uptake and perfusion on whole tumour level, this study will mainly investigate the intra-tumoural relationship between both parameters.

### Data acquisition

Patients underwent [^15^O]H_2_O and [^18^F]FLT PET/CT scans at three different time points: before treatment and 7 and 28 days after start of treatment with a TKI (erlotinib or gefitinib). The type of TKI was chosen by the treating pulmonary physician: 150 mg of erlotinib or 250 mg of gefitinib orally once a day. In the present study, only those patient studies were included for which all three time points were successfully acquired, i.e. at all three time points, both the [^15^O]H_2_O and [^18^F]FLT PET/CT scans were completed.

#### Imaging protocol

The study was performed on a Gemini TF-64 PET-CT scanner (Philips Medical Systems, Cleveland, Ohio, USA). Patients had been fasting 4 h before the start of the scans to avoid possible food-induced thymidine changes. Two venous cannulae were placed, one for tracer injection and the other for blood sampling. The imaging protocol has been described in detail by Frings et al. [3]: first a 10-min dynamic [^15^O]H_2_O scan was performed after intravenous administration of 370 Mbq [^15^O]H_2_O. Next, a low-dose CT scan (50 mAs, 120 kVp) was acquired for attenuation correction of the former scan. This was followed by a 60-min [^18^F]FLT PET scan, after an intravenous bolus injection of 370 Mbq [^18^F]FLT. Afterwards, a second low-dose CT scan was obtained for attenuation correction of the [^18^F]FLT scan.

PET data were normalised and corrected for dead time, randoms, scatter and decay. Data were reconstructed using a vendor provided 3-dimensional row action maximum likelihood iterative reconstruction algorithm (3D RAMLA) with 3 iterations and 33 subsets, a relaxation factor of 1.0, a matrix size of 144 × 144 resulting in voxel sizes of 4 × 4 × 4 mm^3^. These reconstructions were compliant with EANM/EARL specifications. No respiratory gating was performed. The [^15^O]H_2_O scan was reconstructed into 26 frames (1 × 10, 8 × 5, 4 × 10, 2 × 15, 3 × 20, 2 × 30, 6 × 60 s) and the [^18^F]FLT scan into 36 frames (1 × 10, 8 × 5, 4 × 10, 3 × 20, 5 × 30, 5 × 60, 4 × 150, 4 × 300, 2 × 600 s).

#### Blood sampling

Venous blood samples were collected at 5, 10, 20, 30, 40 and 60 min post-injection. Before collecting each sample, 3–5 mL blood was drawn. This was followed by drawing a 7-mL sample and flushing of the cannula with 2.5 mL of saline afterwards. The samples were used for determining the whole-blood and plasma activity concentration and the parent fraction of [^18^F]FLT. These data were used to adjust an image derived (whole blood) input function to acquire a metabolite corrected plasma input function.

### Kinetic analysis

Kinetic analysis was performed using in-house developed software in Matlab (version 7.04; MathWorks Incorporation, Massachusetts, USA). For each lesion, the volume of interest (VOI) was defined on the [^18^F]FLT PET scan. An averaged image of the last three frames of the scan was produced and lesions were delineated using a 50% threshold of the peak standardised uptake value (SUV_peak_).

Image-derived input functions (IDIFs) were generated for both [^15^O]H_2_O and [^18^F]FLT scans, using the early frames in which the first pass of the bolus was best visualised: VOIs of 2 × 2 voxels were positioned in five consecutive axial planes within the lumen of the ascending aorta. These VOIs were then projected onto all image frames to generate a whole blood IDIF. The [^18^F]FLT IDIF was calibrated using the radioactivity concentrations in the venous blood samples. Moreover, IDIFs were corrected for both plasma to whole blood ratios and metabolites to obtain calibrated parent [^18^F]FLT plasma input functions (detailed description in Frings et al. [3]).

Next, parametric images of tumour VOIs were generated. For perfusion (tumour blood flow (TBF) = K_*1*_ of [^15^O]H_2_O), these images were generated using the basis function method [22] and for volume of distribution (V_*T*_) of [^18^F]FLT these images were generated using spectral analysis [23] with optimised settings [24]. Regionally averaged as well as voxel-by-voxel TBF and V_*T*_ data were obtained.

### Association between TBF and [^18^F]FLT V_*T*_

The intra-tumoural association between TBF and [^18^F]FLT V_*T*_ was assessed on a voxel-by-voxel basis by plotting the parametric TBF values against those of [^18^F]FLT V_*T*_.

### Multiparametric classification

A multiparametric classification was applied to TBF and [^18^F]FLT V_*T*_ data of all the lesions, using a global threshold for both parameters. The threshold was determined as the average of all tumour values of all the lesions (for all time points), for TBF and [^18^F]FLT V_*T*_, respectively. First of all, voxels were classified as low or high TBF and low or high V_*T*_ (independently for both parameters), using the threshold (Additional file 1: Figure S1). Voxels with values below the threshold were classified as “low TBF” or “low V_*T*_” whereas voxels with values above the threshold were classified as “high TBF” or “high V_*T*_”. This resulted in the distribution of voxels into two categories for both TBF and V_*T*_ independently (independent classification).

Next, these initial classifications were combined and voxels were classified into four categories (Additional file 1: Figure S1): lowTBF-lowV_*T*_, lowTBF-highV_*T*_, highTBF-lowV_*T*_ and highTBF-highV_*T*_ (multiparametric classification) using the method developed in Iqbal et al. [25]. Depending on the number of voxels in each category, several different voxel distribution patterns could be identified.

### Clustering index

A quantitative metric, the clustering index (CI), was applied to describe the degree of voxel clustering (spatial grouping) within the tumour [26]. The main purpose for using this method was to gain more insight in the spatial coherence/heterogeneity of tumours and the co-localisation of voxels belonging to the same category (e.g. high FLT and high perfusion). The CI was obtained by measuring the local entropy of the classified voxels and was calculated for three (theoretical) situations to determine the expected range for CI per lesion (Additional file 1: Figure S2). By defining this range, effects of tumour volume and shape were taken into account. The following situations were simulated: (1) a condition where all voxels are classified into one category and maximally clustered within the VOI, resulting in a CI of approximately 0; (2) a condition where voxels are classified into four categories and most optimally clustered, providing a CI of approximately 0.3; (3) a condition where voxels are classified in four categories, showing random distribution and almost no clustering, resulting in a CI of approximately 2. Once the range of the CI, given by difference in CI for minimal and maximal clustering, has been determined for each lesion, the actual observed clustering can be expressed as a percentage using the following equation:

1 $$ \mathrm{Clustering}\left(\%\right)=\left(1-\left(\frac{\mathrm{CIlesion}}{\mathrm{CIrange}}\right)\right)\bullet 100 $$

One hundred percent clustering corresponds to the first simulated condition (maximal clustering) whereas 0% clustering corresponds to the third simulated condition (random distribution, no clustering). The CI method has been described and explained in detail by Iqbal et al. [25].

### Statistical analysis

Statistical analyses were performed using SPSS Statistics 20 (IBM Corp.). The correlation between TBF and [^18^F]FLT V_*T*_ (for each time point) was assessed using Pearson’s correlation coefficient. The ANOVA test was performed to assess statistical differences between tumour clustering at the three different time points.

## Results

### Study group

**Fig. 1:**
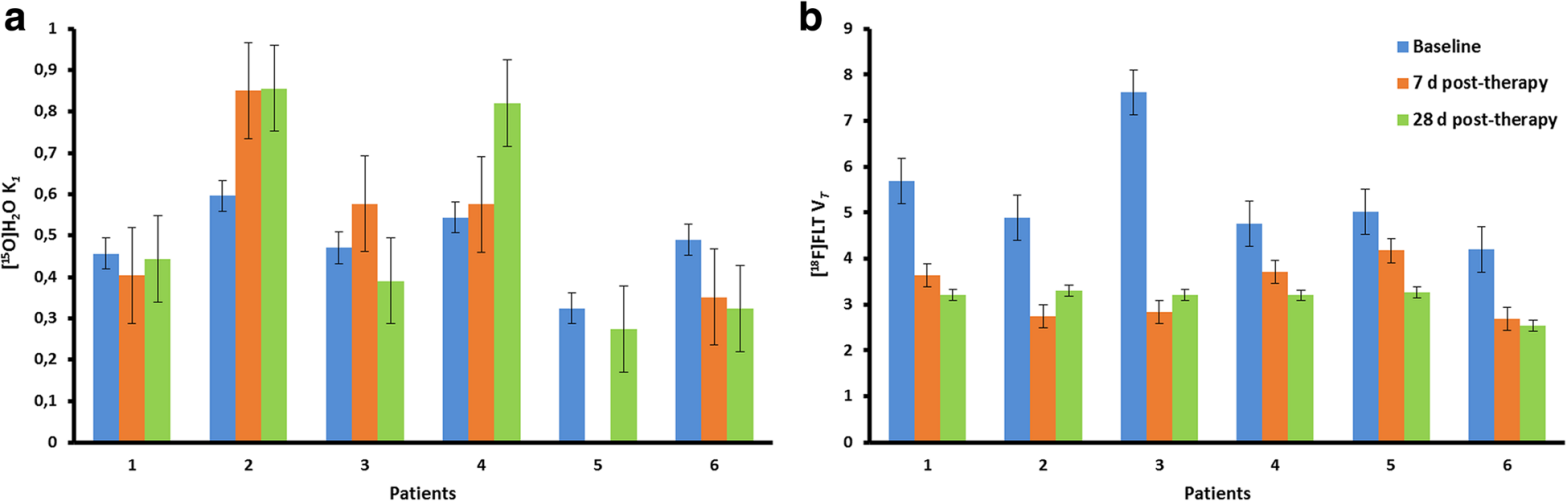
Average TBF (**a**) and [^18^F]FLT V_*T*_ (**b**) values for all the patients across the three time points: baseline, 7 days post-therapy and 28 days post-therapy. The error bars represent standard error

Initially, ten patients were included in this study. However, four patients did not complete imaging studies with both tracers at all time points, and therefore, these four patients were excluded from the study. Finally, the study included six patients with stage IV NSCLC: one male and five females with an average (±SD) age of 65 ± 6 years (Table 1). A total of six malignant lesions, all of which were primary adenocarcinomas, could be identified in the study group (Table 2). The lesions were analysed at three different time points using 17 [^15^O]H_2_O and 18 [^18^F]FLT PET scans. For one patient, data of the [^15^O]H_2_O scan obtained 7 days after starting treatment was unavailable due to technical problems. Average TBF (±SD) values (of all lesions) at baseline and 7 and 28 days after treatment were 0.50 ± 0.21, 0.59 ± 0.31 and 0.54 ± 0.30 mL cm^− 3^ min^− 1^, respectively. Average [^18^F]FLT V_*T*_ (±SD) values (of all lesions) at baseline and 7 and 28 days after treatment were 5.28 ± 1.71, 3.30 ± 1.09 and 3.16 ± 0.66 mL cm^− 3^, respectively. The average lesional values of both parameters are shown in Table 3 and Fig. 1. The global threshold (average ± SD), as used for the multiparametric classification, were 0.53 ± 0.26 mL cm^− 3^ min^− 1^ and 4.25 ± 1.71 mL cm^− 3^ for TBF and [^18^F]FLT V_*T*_, respectively.

**Table 1:** Patient characteristics

All patients (*n*)	6
Male	1
Female	5
Age (years)	
Average	65
Range	57–75
EGFR mutation (*n*)	
Exon 18 G719X	2
Exon 19 Del E746-A750	1
Exon 21 L585R	3
Treatment (*n*)	
Erlotinib	2
Gefitinib	4

**Table 2:** Lesion volumes as assessed on the [^18^F]FLT PET scans at the three time points: baseline, 7 days post-therapy and 28 days post-therapy

	Baseline	7 days post-therapy	28 days post-therapy
Patients	Lesion volume (mL)		
1	46.3	31.2	22.2
2	102.2	45.5	45.4
3	68.3	45.7	68.4
4	126.8	48.9	35.2
5	72.3	32.9	28.9
6	50.8	20.7	19.3

**Table 3:** Average TBF and [^18^F]FLT V_*T*_ values of all the lesions as assessed at the three time points: baseline, 7 days post-therapy and 28 days post-therapy

	Baseline	7 days post-therapy	28 days post-therapy
Patients	TBF		
1	0.46	0.40	0.44
2	0.60	0.85	0.86
3	0.47	0.58	0.39
4	0.54	0.58	0.82
5	0.32	–*	0.27
6	0.49	0.35	0.32
Patients	[^18^F]FLT V_*T*_		
1	5.69	3.63	3.21
2	4.89	2.75	3.30
3	7.62	2.83	3.21
4	4.75	3.71	3.20
5	5.02	4.17	3.26
6	4.20	2.70	2.54

*TBF data for patient 5 was unavailable

### Association between TBF and [^18^F]FLT V_*T*_

**Fig. 2:**
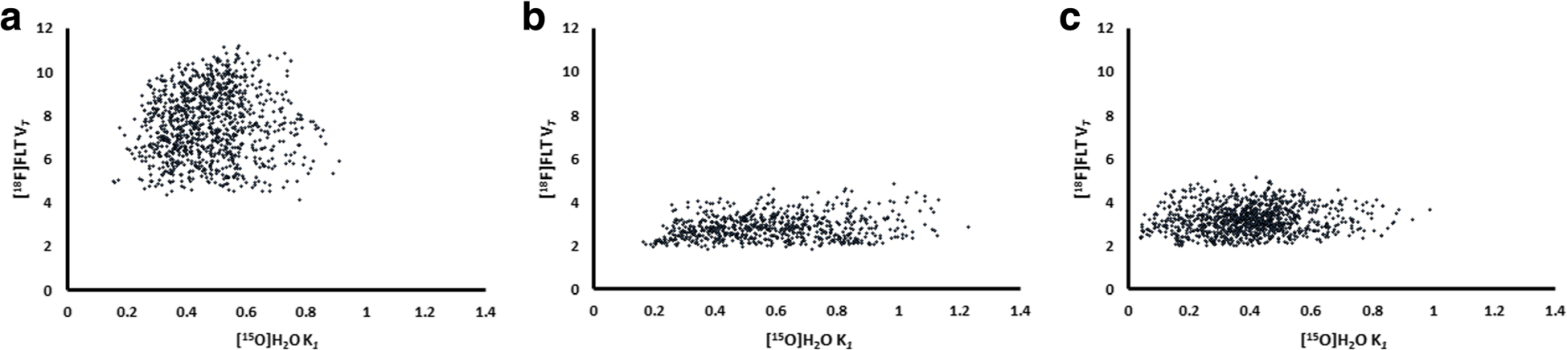
Scatterplots of voxelwise association between TBF and [^18^F]FLT V_*T*_ for the tumour VOI for one patient at the three different time points: baseline (**a**), 7 days post-therapy (**b**) and 28 days post-therapy (**c**)

The association between TBF and [^18^F]FLT V_*T*_, as assessed on a voxel-by-voxel basis, is represented for patient one (Fig. 2). The association was assessed at three different time points: baseline (Fig. 2a), 7 days after treatment (Fig. 2b) and 28 days after treatment (Fig. 2c). Pearson’s correlation showed no significant relationship between TBF and [^18^F]FLT V_*T*_ (baseline, *r* − 0.14, *P* = 0.79, 7 days after treatment, *r* − 0.24, *P* = 0.69, 28 days after treatment, *r* 0.41, *P* = 0.43). At all the three time points, data were scattered over a large range of TBF values. A specific trend, such as decreased tracer uptake with decreased TBF, could not be observed. Over the three time points, a clear decrease in [^18^F]FLT V_*T*_ could be perceived which was most prominent comparing the data at baseline with the data obtained 7 days after treatment. However, such a trend could not be identified for TBF.

### Multiparametric classification

**Fig. 3:**
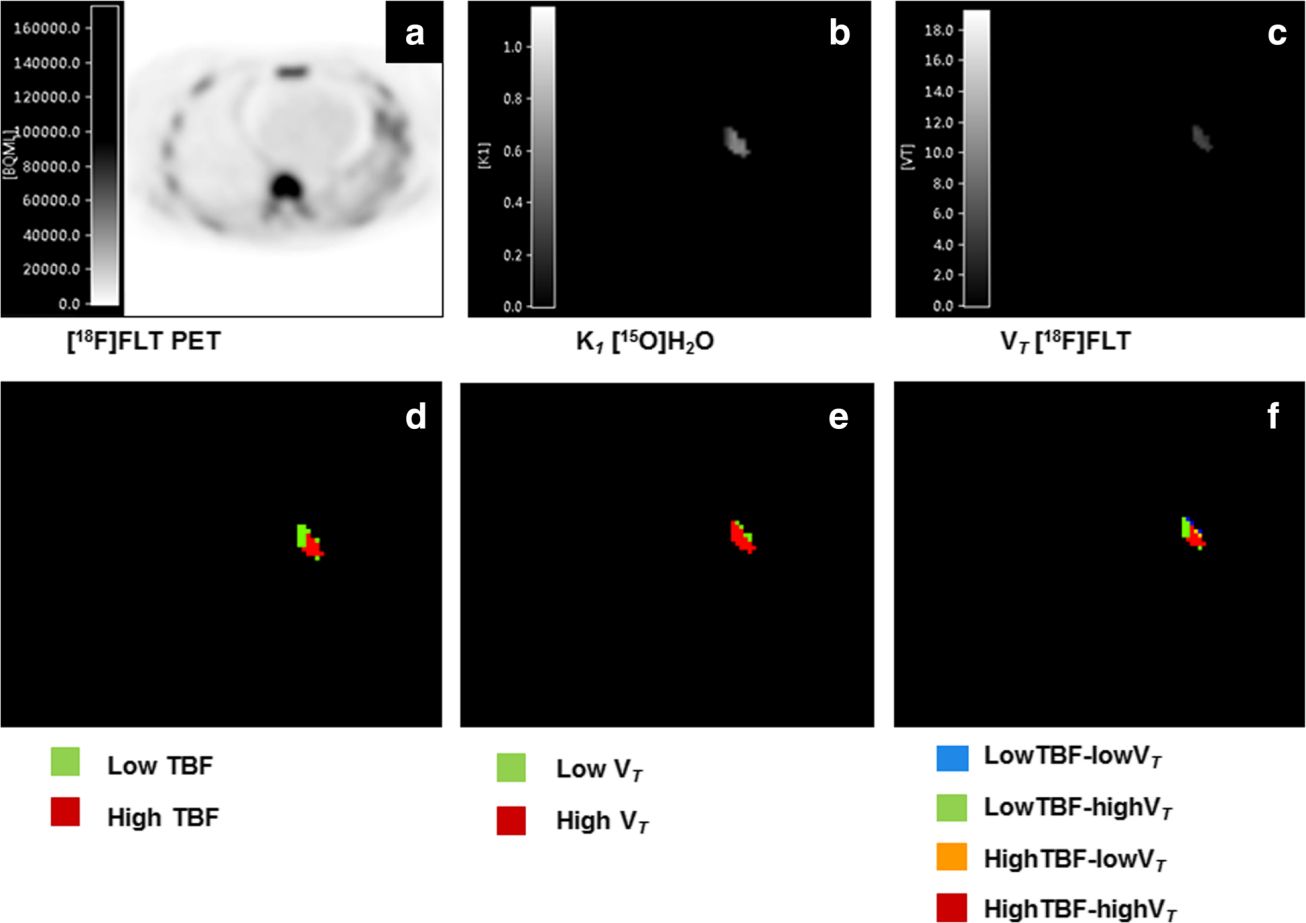
An overview of the multiparametric evaluation of TBF and [^18^F]FLT uptake. **a** [^18^F]FLT PET scan. **b** Parametric VOI image of [^15^O]H_2_O derived TBF. **c** Parametric VOI image of [^18^F]FLT V_*T*_. **d** Voxels classified as low or high TBF. **e** Voxels classified as low or high [^18^F]FLT V_*T*_. **f** Voxels classified into four categories: lowTBF-lowV_*T*_, lowTBF-highV_*T*_, highTBF-lowV_*T*_ and highTBF-highV_*T*_

**Fig. 4:**
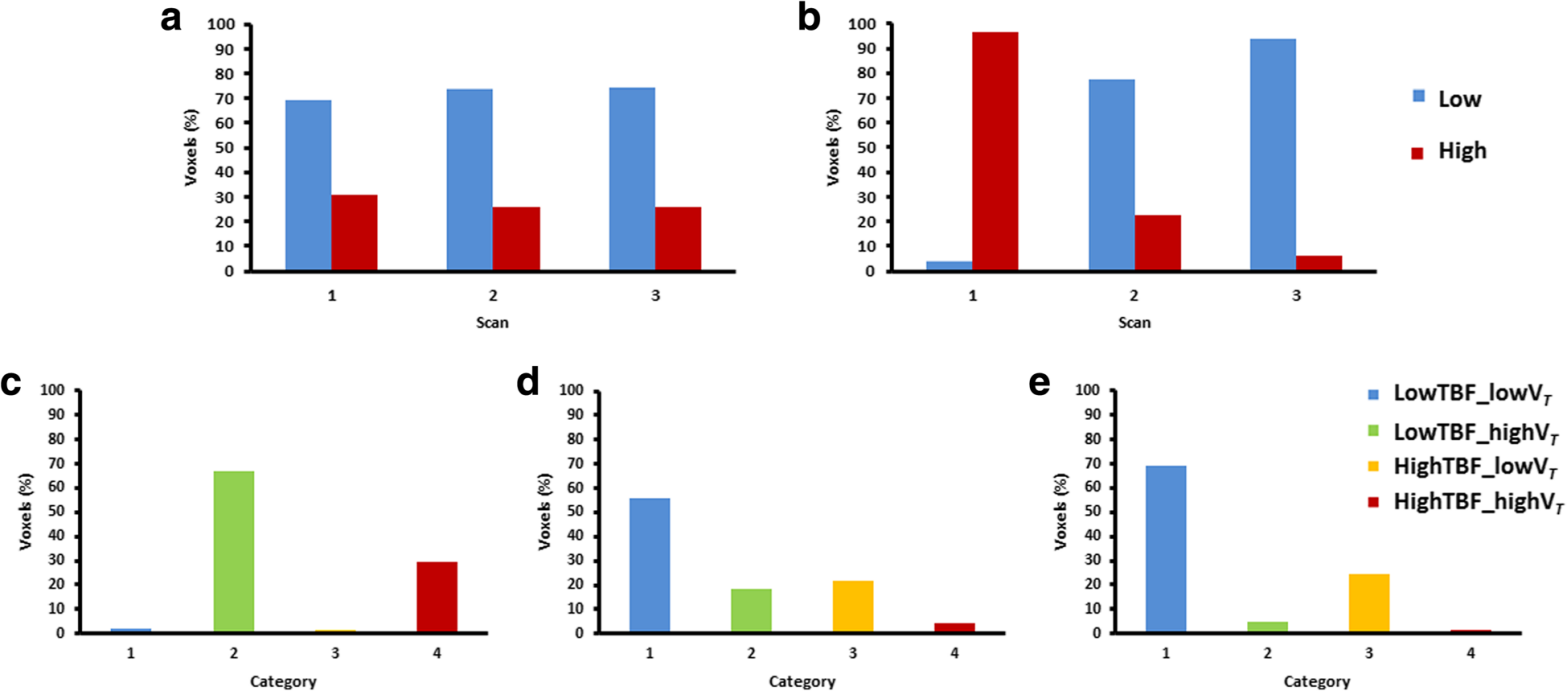
Independent (**a**, **b**) and multiparametric (**c**, **d**, **e**) classification of one lesion. The independent classification shows the percentage of voxels classified in the “low” or “high” category for TBF (**a**) and [^18^F]FLT V_*T*_ (**b**), for the three time scan time points: baseline (1), 7 days post-therapy (2) and 28 days post-therapy (3). The multiparametric classification shows the percentage of voxels in each category of the classification, for all the three time points: baseline (**c**), 7 days post-therapy (**d**) and 28 days post-therapy (**e**). Voxels are classified into four categories using the global threshold

TBF and [^18^F]FLT V_*T*_ data were evaluated using the independent and multiparametric classification (Fig. 3). Figure 4 represents the voxel distribution patterns for one patient, for both the classifications. The overall evaluation for all patients revealed that across the three time points, changes in TBF were minimal. However, in comparison to TBF, [^18^F]FLT V_*T*_ underwent a shift in voxel distribution over the three time points. At baseline, most voxels were classified in the “highV_*T*_” (average 77.1%, range 47.4–99.9%) or “lowTBF-highV_*T*_ (average 45.5%, range 14.1–70.1%) and highTBF-highV_*T*_ (average 31.5%, range 2.7–59.7%)” category, depending on the classification applied (independent or multiparametric). One week after start of the therapy, most voxels were classified in the “lowV_*T*_” (average 85.4%, range 65.0–100%) or “lowTBF-lowV_*T*_ (average 47.7%, range (18.4–90.4%) and highTBF-lowV_*T*_” (average 41.9%, range 9.6–73.8%) category. This distribution pattern remained similar afterwards.

Visual inspection of the voxel distribution showed that voxels classified within one category were clustered. Tumours could be visually delineated into several different regions, depending on its TBF and [^18^F]FLT V_*T*_. Voxels in the “lowTBF-lowV_*T*_” category were mainly located at the tumour edges whereas voxels in the “highTBF-highV_*T*_” category could often be identified at the core of the tumour. Voxels in the remaining categories showed a more dispersed distribution pattern across the lesions.

### Clustering index

**Fig. 5:**
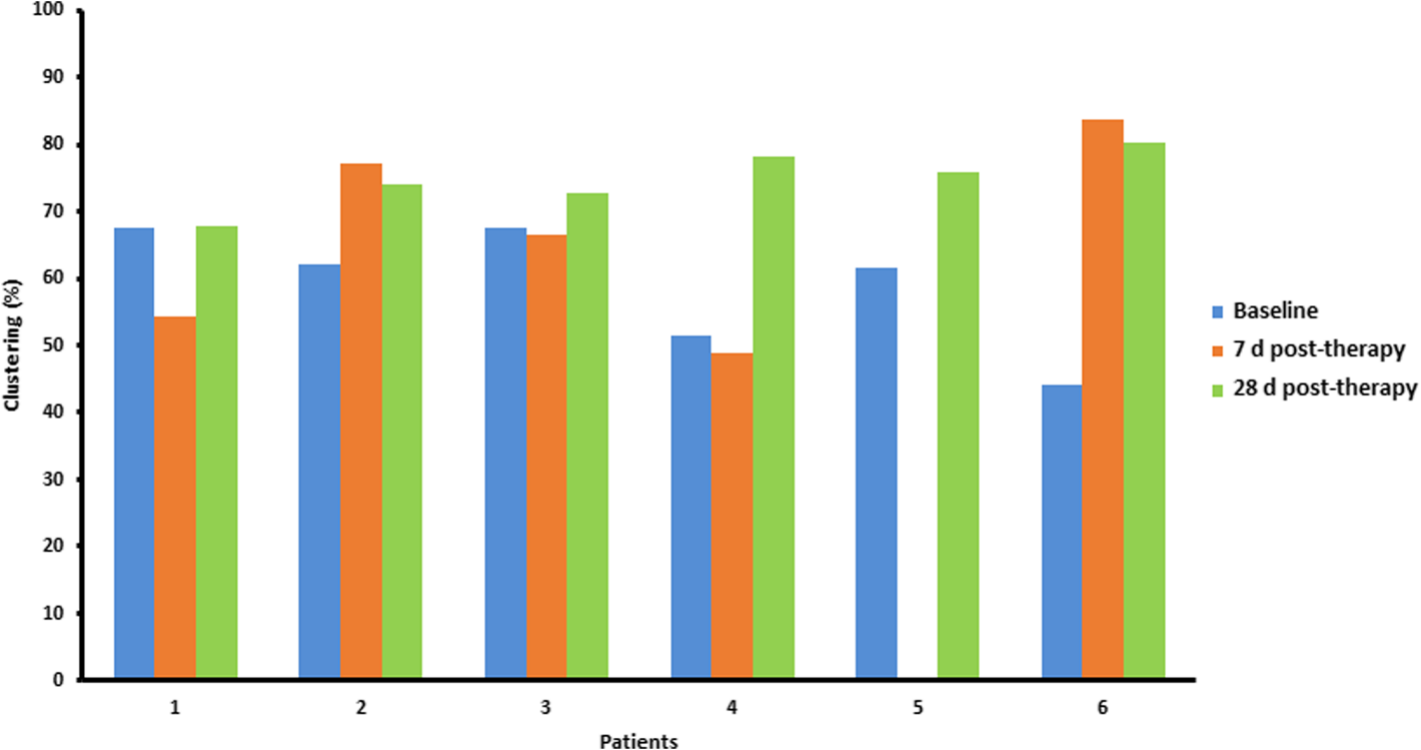
Clustering (%) represents the degree of voxel clustering for all lesions, for the three time points: baseline, 7 days post-therapy and 28 days post-therapy. Voxels are more clustered when the percentage is closer to 100%. *TBF data for patient 5 was unavailable and therefore the clustering parameter could not be determined 7 days after treatment

Quantitative analysis using the CI also showed that voxels were clustered rather than randomly distributed across the lesions. It indicated that the degree of clustering was significantly different from 0% (*P* < 0.001) for all three time points. Table 4 represents clustering (%) for all lesions at the three different time points (average ± SD; baseline 59.1% ± 9.4%, 7 days post-therapy 66% ± 14.7, 28 days post-therapy 74.8% ± 4.4%). It shows an increase in clustering when comparing data at baseline with data obtained 28 days after treatment (Fig. 5; ANOVA test, *P* = 0.016).

**Table 4:** Clustering (%) represents the degree of voxel clustering for all lesions, for the three time points: baseline, 7 days post-therapy and 28 days post-therapy. Voxels are more clustered when the percentage is closer to 100%

Patients	Clustering (%)		
	Baseline	7 days post-therapy	28 days post-therapy
1	67.8	54.3	67.8
2	62.1	77.1	73.9
3	67.4	66.7	72.7
4	51.5	49.0	78.2
5	61.6	–*	75.8
6	44.2	83.8	80.3

*TBF data for patient 5 was unavailable and therefore the clustering parameter could not be determined

## Discussion

### Association between TBF and [^18^F]FLT V_*T*_

We hypothesised that TBF could affect tracer uptake: low perfused areas might show low tracer uptake due to perfusion-limited delivery of [^18^F]FLT. However, our data reveals that TBF does not seem to affect tracer uptake, as assessed with scatterplots (Fig. 2) and the classifications/cluster index applied. The [^18^F]FLT V_*T*_ is comparable in low and high perfused areas and does not correlate with TBF. In addition, changes in [^18^F]FLT V_*T*_ after start of treatment do not seem to accompany the (minimal) changes in TBF. This might indicate that [^18^F]FLT distribution and its changes do not depend on perfusion which is a preferable characteristic of the [^18^F]FLT PET tracer.

### Multiparametric evaluation

Prior to the multiparametric evaluation, TBF and [^18^F]FLT V_*T*_ data were evaluated independently. For TBF, most voxels were classified in the “lowTBF” category and this distribution pattern persisted over the three time points. As patients were treated with erlotinib or gefitinib a decrease in TBF was expected. The administered TKIs influence the VEGF signalling pathway through the EGFR pathway. In healthy tissue, activation of the EGFR pathway leads to production of VEGF, the primary inducer of angiogenesis [19, 21]. However, during TKI treatment, both pathways are inhibited. This results in decreased angiogenesis, eventually resulting in decreased TBF and tumour necrosis. De Langen et al. monitored response to antiangiogenic therapy (bevacuzimab and erlotinib) by investigating changes in TBF [9]. They assessed that mean TBF decreased by 11% 3 weeks after starting treatment. Another study also showed a decrease in TBF 2 weeks after treatment with Sunitinib [27]. Mankhoff et al. investigated changes in TBF in patients receiving neoadjuvant chemotherapy [28]. They differentiated between responders and non-responders, the first showing a decrease in TBF whereas the latter showed an increase in TBF. Although our study does not show distinct changes in TBF, it might be possible that these changes occur at a later stage of treatment as angiogenesis and perfusion changes are relatively slow processes.

Furthermore, our data shows that [^18^F]FLT V_*T*_ decreases after start of TKI treatment with the most prominent decrease observed 7 days after treatment. Kahraman et al. also presented similar results in NSCLC patients, showing decreased [^18^F]FLT uptake in early PET images (1 week after starting treatment with erlotinib) [29]. They concluded that early evaluation with [^18^F]FLT PET is better than late evaluation (6 weeks after treatment with erlotinib) for response prediction and prediction of progression-free survival. Several other studies also showed a decrease in [^18^F]FLT uptake 1 or 2 weeks after therapy, which correlated with progression-free survival [12, 30]. Our study also evaluated survival data to assess a possible relationship between overall survival and the voxel distribution patterns of the multiparametric classification. However, no such relationship could be found, possibly due to the small population size.

In addition, the visual analysis suggested that voxels classified within one category were clustered rather than randomly distributed across the lesions. It was possible to visually delineate a tumour into several different regions, depending on the voxel distribution of TBF and [^18^F]FLT V_*T*_, independently and multiparametric. Voxels in the “lowTBF-lowV_*T*_” category were mainly located at the tumour edges; this might be a partial volume effect: due to tumour movement caused by respiration, the PET signal might be blurred, therefore showing a low PET signal [25]. On the contrary, voxels in the “highTBF-highV_*T*_” category could often be identified at the core of the tumour, indicating high angiogenesis and proliferation in the core of the tumour [25]. The voxel distribution varied intralesionally as well as interlesionally, indicating spatial heterogeneity of these tumours [31, 32]. This can be the result of heterogeneity at genetic and cellular levels as well as at a tissue level [33–35]. The first refers to mutational events, which take place during tumour development. These mutations might vary between tumours, which might be one of the mechanisms responsible for differences in response to treatment and resistance to treatment. The heterogeneity at the tissue level indicates differences in vascular density, blood flow, regional metabolism and proliferation. This study assessed the diversity of TBF and proliferation rate within NSCLC tumours and showed that each tumour has its own heterogeneity.

### Limitations and scientific implications

This study has certain limitations, and one of these includes the small sample size (*n* = 6, with each three scans). To increase the reliability of the study performed, it would be recommended to perform it with a larger sample size in future. Secondly, as during this study patients with lung cancer were scanned (scanning field: thorax), respiratory motion might have led to imaging artefacts (this might have affected further analysis and the results) or reduced spatial resolution. However, the presence of respiratory motion-related artefacts was carefully checked and these were not visually identified. Moreover, the primary tumours in our study were relatively large (≥ 3 cm diameter), and for these tumours, the tracer uptake is usually less affected by motion. Yet, to limit the presence of respiratory motion artefacts, respiratory gating might be a helpful technique for future studies. Finally, while performing the kinetic analysis, lesions on PET scans were delineated using the 50% SUV_peak_ threshold. This might have led to exclusion of low perfused and/or low tracer uptake areas. However, during our study, visual inspection of the VOI using both PET and CT data did not suggest that tumour tissue was missed and this threshold could be used.

In our study, we evaluated the relationship of tumour perfusion and [^18^F]FLT tracer uptake responses in patients suffering from adenocarcinoma and treated with EGFR TKIs. We found that [^18^F]FLT responses occurred independent from perfusion (changes), which suggests that [^18^F]FLT uptake and its change may be considered as a unique biomarker independent from perfusion. The authors believe that these results are generally applicable to other types of NSCLC patients and therapies. However, for other patients and therapies, a treatment response or change in both perfusion and [^18^F]FLT uptake might occur. Our results, however, suggest that [^18^F]FLT uptake changes as a response marker can be observed regardless of change in perfusion.

[^15^O]H_2_O and [^18^F]FLT PET are a non-invasive method for the assessment of perfusion and cellular proliferation, respectively. The acquired data was used for generating the multiparametric classification. As it is a new method for analysing changes in perfusion and tracer uptake, further evaluation of this method is necessary to reliably apply this in future studies. Clinical data, such as resection specimens, could be useful for the assessment of perfusion and proliferation (using immunohistochemical staining). It would be interesting to compare pathology with the results of the multiparametric classification and investigate similarities or differences. It would also be interesting to assess perfusion and proliferation at a later stage of treatment. During the follow-up of this study, changes in perfusion were limited, while changes in proliferation were prominent. It might be interesting to see how this evolves when patients are under treatment for a longer time as in that case changes in perfusion might occur or become more prominent as angiogenesis and perfusion changes are relatively slow processes.

## Conclusion

The present study suggests that [^18^F]FLT might be a perfusion-independent PET tracer for measuring tumour response as parametric changes in [^18^F]FLT uptake occurred independent from changes in perfusion.

## Additional file

Additional file 1: **Figure S1.** A representation of different voxel distribution patterns. The presented distributions are hypothetical. The independent classification consists of voxels classified in two categories, low and high, for TBF and [^18^F]FLT V_*T*_ independently (independent classification). A combination of these classifications results in the multiparametric classification, where voxels are classified into four categories: lowTBF-lowV_*T*_ (category 1), lowTBF-highV_*T*_ (category 2), highTBF-lowV_*T*_ (category 3), highTBF-highV_*T*_ (category 4). **Figure S2.** A representation of three possible patterns of voxel clustering. (a) Pattern which represents maximal clustering. (b) Pattern which represents optimal clustering with voxels classified in four different categories. (c) Pattern which represents random distribution of voxels classified in four different categories. (DOC 215 kb)

## Abbreviations

[^18^F]FLT: 3′-Deoxy-3′-[^18^F]fluorothymidine
3D RAMLA: 3-Dimensional row action maximum likelihood reconstruction algorithm
CI: Clustering index
EGFR: Epidermal growth factor receptor
IDIF: Image-derived input function
NSCLC: Non-small cell lung cancer
PET: Positron emission tomography
SUV_peak_: Peak standardised uptake value
TBF: Tumour blood flow
TKI: Tyrosine kinase inhibitor
VEGF: Vascular endothelial growth factor
VOI: Volume of interest
V_*T*_: Volume of distribution
