# Supplementary material for: Validation of [18F]FLT as a perfusion-independent imaging biomarker of tumour response in EGFR-mutated NSCLC patients undergoing treatment with an EGFR tyrosine kinase inhibitor
Source: EJNMMI Res. 2018 Mar 27;8:22. doi: 10.1186/s13550-018-0376-6 (PMC5874225; doi:10.1186/s13550-018-0376-6)
Supplement: Supplementary file 1 — Figure S1. A representation of different voxel distribution patterns. The presented distributions are hypothetical. The independent classification consists of voxels classified in two categories, low and high, for TBF and [18F]FLT VT independently (independent classification). A combination of these classifications results in the multiparametric classification, where voxels are classified into four categories: lowTBF-lowVT (category 1), lowTBF-highVT (category 2), highTBF-lowVT (category 3), highTBF-highVT (category 4). Figure S2. A representation of three possible patterns of voxel clustering. (a) Pattern which represents maximal clustering. (b) Pattern which represents optimal clustering with voxels classified in four different categories. (c) Pattern which represents random distribution of voxels classified in four different categories. (DOC 215 kb) [file 13550_2018_376_MOESM1_ESM.doc]

**Additional file 1**

**
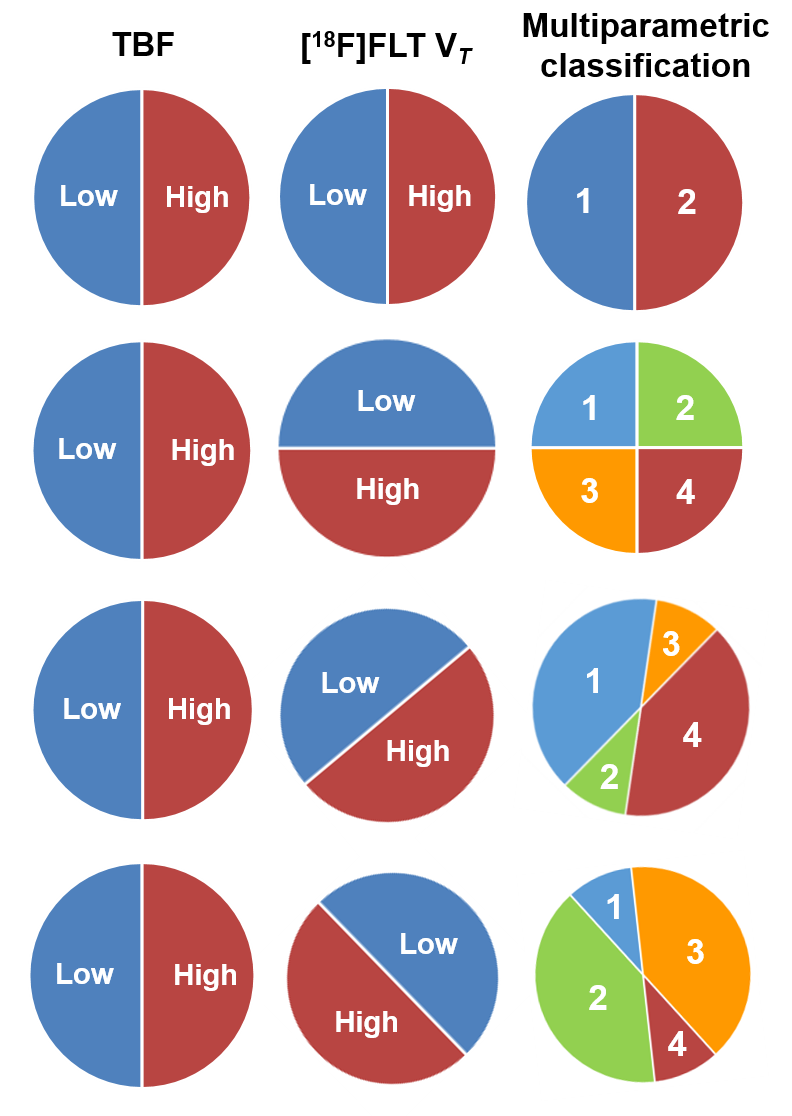
**

**Figure S1** A representation of different voxel distribution patterns. The presented distributions are hypothetical. The independent classification consists of voxels classified in 2 categories, low and high, for TBF and [^18^F]FLT V*_T_* independently (independent classification). A combination of these classifications results in the multiparametric classification, where voxels are classified into 4 categories: lowTBF-lowV*_T_* (category 1), lowTBF-highV*_T_* (category 2), highTBF-lowV*_T_* (category 3), highTBF-highV*_T_* (category 4).


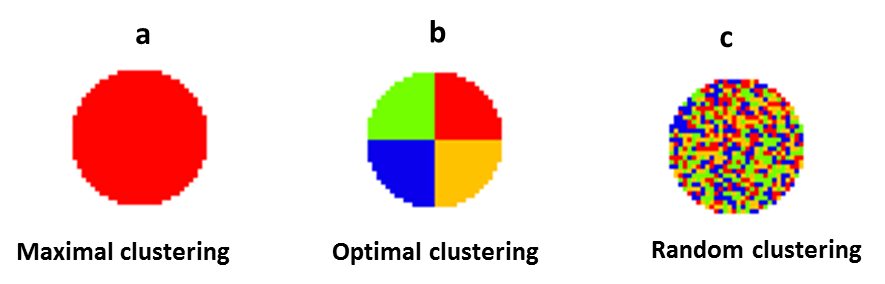


**Figure S2.** A representation of three possible patterns of voxel clustering. (a) Pattern which represents maximal clustering. (b) Pattern which represents optimal clustering with voxels classified in 4 different categories. (c) Pattern which represents random distribution of voxels classified in 4 different categories.
